# Synthesis of Cyclic Fragrances via Transformations of Alkenes, Alkynes and Enynes: Strategies and Recent Progress

**DOI:** 10.3390/molecules27113576

**Published:** 2022-06-02

**Authors:** Zhigeng Lin, Baoying Huang, Lufeng Ouyang, Liyao Zheng

**Affiliations:** School of Chemistry and Chemical Engineering, Guangzhou University, Guangzhou 510006, China; 2111905017@e.gzhu.edu.cn (Z.L.); 1805100021@e.gzhu.edu.cn (B.H.); 2111805015@e.gzhu.edu.cn (L.O.)

**Keywords:** cyclization, cycloaddition, carbocycles, heterocycles, unsaturated hydrocarbons, natural fragrances, synthetic odorants, transition-metal catalysis, asymmetric organocatalysis, cascade/tandem/domino reaction

## Abstract

With increasing demand for customized commodities and the greater insight and understanding of olfaction, the synthesis of fragrances with diverse structures and odor characters has become a core task. Recent progress in organic synthesis and catalysis enables the rapid construction of carbocycles and heterocycles from readily available unsaturated molecular building blocks, with increased selectivity, atom economy, sustainability and product diversity. In this review, synthetic methods for creating cyclic fragrances, including both natural and synthetic ones, will be discussed, with a focus on the key transformations of alkenes, alkynes, dienes and enynes. Several strategies will be discussed, including cycloaddition, catalytic cyclization, ring-closing metathesis, intramolecular addition, and rearrangement reactions. Representative examples and the featured olfactory investigations will be highlighted, along with some perspectives on future developments in this area.

## 1. Introduction

Fragrances, short for fragrance ingredients, is a type of compounds with a sweet smell or pleasant odor that has wide applications in the fine chemical industry, especially in perfumes, cosmetics, detergents and food additives [1,2,3,4]. The perception of aroma and odor is a subjective phenomenon that is more difficult to describe precisely and measure quantitatively than the perception of light or sound [5,6,7,8,9,10,11,12]. Thus, fragrances with different structures are highly desirable for yielding various scents with repeated effects and long shelf life; they are key to studying the principle of olfaction, including the range and resolution of the olfactory system and the working mechanism of odorant receptors [2,5,6,7,8,9,10,11,12,13,14,15,16,17].

In ancient times, fragrances were mainly obtained from natural resources such as plant essential oils and animal secretions [3,4,18,19]. While natural ingredients still represent an important class of fragrances, they cannot meet the rapidly growing and diversified needs of consumers and perfumers. The power of organic synthesis for fragrances was unlocked in 1868 when coumarin was successfully prepared by W. H. Perkin from salicylaldehyde. With the blossoming of organic synthetic methods and the related industrial technologies over the past century, synthetic and semi-synthetic fragrances emerged to become mainstream in the flavors and fragrances industries [2,18,19,20,21,22,23,24,25,26,27,28,29,30,31,32,33,34,35,36]. Nowadays, fragrance molecules represent an attractive testing ground and offer a multi-billion-dollar outlet for organic synthesis, stimulating researchers in academia and fragrance manufacture to seek out new fragrances and explore more efficient, economical, scalable and environmentally friendly synthetic methods.

**Figure 1:**
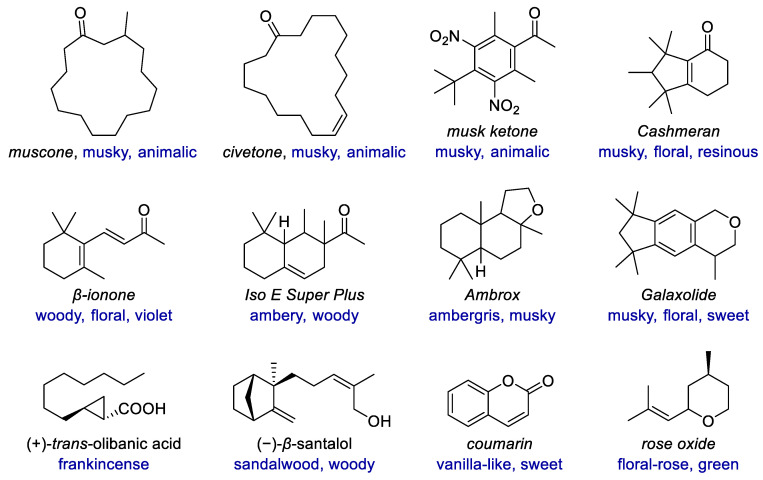
Examples of fragrances with diverse carbocyclic or heterocyclic scaffolds.

Similar to medicinal chemistry, some structures have been established as privileged structures in fragrance chemistry, many of which have one or more carbo- or heterocycles (Figure 1). The ring sizes range from that of cyclopropane in olibanic acid to the macrocycles in muscone and civetone. Diverse types of rings, including carbocyclic and heterocyclic, saturated and unsaturated, aromatic and non-aromatic, monocyclic and polycyclic rings exist in both natural and synthetic fragrances. With highly structural diversity and the resulting tunable odor characters, cyclic fragrances have emerged as molecular probes for exploring olfactory chemical space. New synthetic methods enable much more structural diversity of fragrances, including the scaffold diversity of different rings and their linking modes, as well as the variations of substituents and their replacement by heteroatoms. These compounds include not only derivatives and analogs of natural fragrances but also those compounds with novel scaffolds due to rational design or unexpected findings.

Unsaturated hydrocarbons, especially alkenes and alkynes, are extensively used for the construction of carbo- and heterocycles due to their adjustable reactivity and cyclization modes. Many of these building blocks can be easily obtained from natural resources or petrochemicals via reliable processes. In addition, the reactions involving unsaturated bonds usually have high atom economy. With classic or new odorants as targets, innovative synthetic strategies and methods have been developed recently that enrich the frontiers of alkene and alkyne chemistry. Though there are a few reviews published on fragrance synthesis [2,18,19,20,21,22,23,24,25,26,27,28,29,30,31,32,33,34,35,36], the importance of unsaturated hydrocarbons in this area is still underestimated, especially that of alkynes and enynes.

In this review, several widely used or newly emerged synthetic methods for the production of cyclic fragrances will be introduced, with an emphasis on the transformation of carbon-carbon double/triple bonds. Both the total synthesis of natural fragrances and the discovery of new odorants will be discussed, along with their featured olfactory studies. The key ring-forming steps and selected products with their odor descriptions will also be illustrated in the form of schemes. For the synthetic methods, new cyclization modes and strategies, transition-metal or organocatalyzed transformations, regioselectivity or stereoselectivity, scaffold and heteroatom diversity, as well as improvements in green chemistry and sustainability, will be highlighted.

## 2. Background of the Olfactory System and Fragrance Chemistry

Pondering the mystery of olfactory perception can be dated back to ancient times. As an extension of Democritus’ atom theory, the sweet odorant is proposed as being made up of atoms with smooth surfaces, while the acidic odorant is made up of atoms with sharp points, which have different effects upon contact with the nose. Since the landmark work of R. Axel and L. Buck in 1991 [37], who discovered a diverse superfamily of genes that encodes odorant receptors, the molecular mechanism of the olfactory system has been widely investigated [5,38,39,40,41,42,43,44,45,46,47,48]. The odorant receptors are expressed on the surface of olfactory receptor neurons (ORNs) in the olfactory epithelium and comprise the largest family of G-protein-coupled receptors (GPCRs). The olfactory epithelium is a type of membranous tissue in the nasal cavity that consists of three types of cells, including ORNs, supporting cells and basal cells. When a set of olfactory receptors are activated by odorants, the corresponding ORNs first send neural signals through their axons to a specific glomerulus in the olfactory bulb, then to the central neurons.

Although mammals have developed hundreds to thousands of odorant receptors, a combinatorial strategy is necessary to recognize about ten thousand different odors. Though an ORN typically expresses only one type of olfactory receptor, an odorant receptor can be activated by many odorants, and most odorants can activate several or dozens of receptors. Odorant receptors are able to recognize different features of molecules, such as their functional group, molecular shape and length, ring size, substitutions, chirality, polarity and vibrations. Besides its perfect match with a single odorant receptor, an odor molecule may partially activate several other receptors. Thus, the recognition of an odor molecule is not the activation of an on/off switch but is instead a process to generate an odorant response pattern with an array of receptors that activate to a different extent.

Although a brief blueprint of the molecular basis of the olfactory system has been unveiled, an in-depth and detailed understanding of the mechanism is still to be developed. Compared to other types of GPCRs, the ligands of olfactory receptors are much less widely studied. Some fragrances, both natural and synthetic ones, have been proved or proposed to be the ligands of specific olfactory receptors and are related to a range of biological activities. For example, the olfactory receptor OR5AN1 in humans and MOR215-1 in mice can respond to macrocyclic musks such as muscone [49,50]. *β*-Ionone can activate the olfactory receptor OR51E2 and regulates a series of activities in the cell [51,52]. However, the structure–odor relationships may be more confusing in the case of structure–activity relationships for drugs, due to the combinatorial mode for odorant recognition.

In the well-known Michael Edwards fragrance wheel, fragrances are divided into four major classes with fresh, floral, ambery and woody notes, respectively, each of which is further divided into several subclasses, including the transition zones between two classes. In another classification, fragrance characters are divided into eight classes ranging from fruity, green, marine, floral, spicy, woody, ambery, musky, and then back to fruity to form a cyclic spectrum. These olfactory spectra resemble the color spectrum, but there is no simple parameter or fundamental rule similar to the wavelengths and frequencies of light that have been found so far. Nowadays, although fragrance chemists and perfumers have a large library of fragrances from which to deliver a wide spectrum of scents, it is still challenging to predict the odor of a molecule and to design a molecule with a specific odor. We anticipate that the synthetic advances illustrated below will enrich the synthetic toolkit, helping researchers to devise new fragrances and better ligands for olfactory receptors, and will encourage more organic chemists to become interested in fragrances.

## 3. Synthesis of Fragrances via Cycloaddition or Formal Cycloaddition

**Scheme 1:**
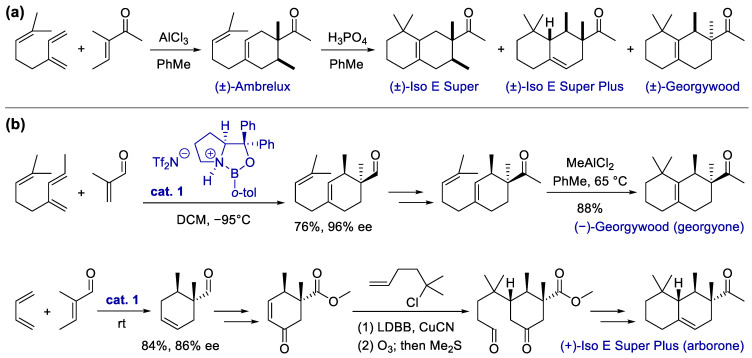
Synthesis of carbocyclic fragrances via the Diels–Alder reaction. (**a**) Synthesis of Ambrelux and its conversion via acid-promoted annulative rearrangement; (**b**) total synthesis of georgyone and arborone, with the enantioselective Diels–Alder reaction as a key step.

Since the discovery of the Diels–Alder reaction, the cycloaddition of π reactants serves as one of the most powerful methods for the construction of carbocycles, which has a broad application in the fragrance industry [53]. One classic example is the production of Ambrelux from myrcene via the AlCl_3_-promoted Diels–Alder reaction [33] (Scheme 1a). Some of the attractive features of cycloaddition include perfect atom economy up to 100%, good functional group compatibility due to typically redox-neutral conditions, and the modular ability to access products via multiple substituents from readily available building blocks.

Traditional cycloadditions under heating, irradiation or Lewis–acidic conditions are widely used in both laboratory synthesis and industrial fragrance manufacture. In this section, we would like to focus on cyclizations with an organo- or metal-catalyzed process, especially those with unique stereochemical control or cyclization modes. A remarkable example is reported by Hong and Corey, who developed an elegant enantioselective total synthesis of georgyone and arborone [54] (Scheme 1b). The key ring-forming and enantioselective step shared by both routes is a chiral oxazaborolidinium cation-catalyzed Diels–Alder reaction using the corresponding 1,3-butadienes and butenals. The double bond in the adducts facilitates further transformations, including a final annulative isomerization to furnish georgyone and an accelerated 1,4-addition, followed by ozonolysis, to give the keto–aldehyde intermediate toward the production of arborone.

Georgyone and arborone are bicyclic fragrances with intense, clean and transparent woody-ambery note. Interestingly, the name “arborone” (from the Latin word *arbor*) was first proposed by Corey et al. in their paper, as they found this enantiomer is the key ingredient and deserved an independent term. The racemic form was previously named as “Iso E Super Plus”, which was found unexpectedly as an impurity in Iso E Super that synthesized by an acid-promoted cyclization of Ambrelux [33] (Scheme 1a). Later, another isomeric side product was identified and patented by Givaudan as Georgywood. The odor threshold of (−)-Georgywood was found to be more than 100 times greater than its antipode [55], which stimulated Corey et al. to propose an exclusive term, “georgyone”. Besides these two ingredients, Corey et al. further synthesized several analogs and performed a binding analysis to clarify structural features of woody odorants, including absolute configuration, methyl substitution and spatial orientation. Their work revealed the “magic methyl effect” on this privileged scaffold and opened up an avenue for studying the related olfactory receptors and spatial organization of olfactory glomeruli.

**Scheme 2:**
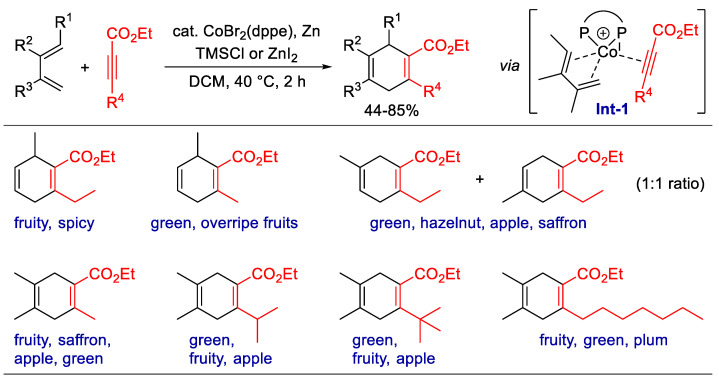
Synthesis of fragrant 1,4-cyclohexadiene carboxylates via Co-catalyzed [4 + 2] cyclization.

The six-membered ring ester is an attractive scaffold for fragrances with fruity and floral odors, as exemplified by the well-known ethyl safranate. Their odor characteristics have been proven to relate to the double bonds and substituents, but the study of 1,4-cyclohexadiene carboxylates is underdeveloped due to a lack of practical synthetic methods. In 2020, Goeke et al. reported the Co-catalyzed formal [4 + 2] cycloaddition of 1,3-dienes and alkyl propiolates [56] (Scheme 2). The obtained 1,4-cyclohexadiene carboxylates typically demonstrate fruity and green odors, which vary according to their substituents. However, this catalytic system cannot control the regioselectivity of unsymmetrical dienes and unseparated mixture of two regioisomers was obtained.

In the proposed catalytic cycle, the in situ formed Co(I) species is coordinated by the diene and alkyne sequentially to form **Int-1**, which may undergo metallocyclization or follow direct [4 + 2] cycloaddition pathways to form the adduct. The design and screening of ligands would make it possible to achieve regioselectivity or even enantioselectivity. Recently, Shi et al. developed an enantioselective [4 + 2] cycloaddition, catalyzed by a Rh(I) complex with a chiral phosphoramidite ligand [57]. The reaction can be efficiently scaled up by using 26.2 g (E)-1,3-nonadiene and 10 g dimethyl acetylenedicarboxylate to yield the product at 99% ee in 92% yield. However, less active propiolates, such as methyl 2-butynoate, are totally inactive in this reaction, which limits their application for the synthesis of fragrances. Therefore, advanced catalytic systems still need to be developed for the synthesis of fragrant 1,4-cyclohexadienes, with a focus on the improvement of both activity and regioselectivity for unsymmetrically substituted substrates.

**Scheme 3:**
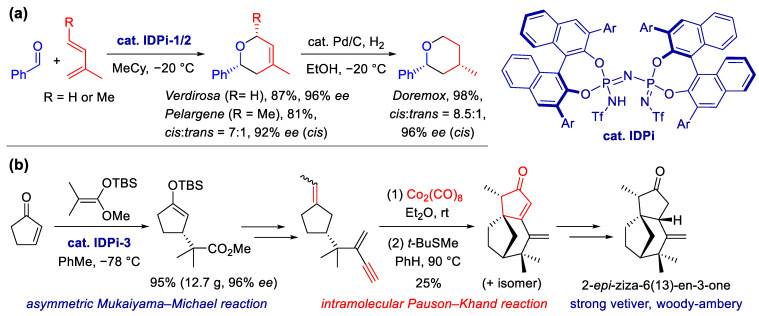
Fragrance synthesis by the List group. (**a**) Synthesis of hydropyran fragrances via the asymmetric hetero-Diels–Alder reaction, catalyzed by imidodiphosphorimidate (IDPi). (**b**) Total synthesis of the principal ingredient of vetiver oil with IDPi-catalyzed asymmetric Mukaiyama–Michael addition and Co_2_(CO)_8_-mediated Pauson–Khand [2 + 2 + 1] cyclization as the key steps.

Organocatalysis is a powerful toolkit for the construction of C–C bonds and rings via enantioselective addition or cycloaddition. The List group has developed a new type of Brønsted acid catalysts, imidodiphosphorimidates (IDPis), which have a rather high acidity and a confined chiral microenvironment [58]. In 2017, List et al. reported an asymmetric hetero-Diels–Alder reaction between dienes and aldehydes to furnish multi-substituted dihydropyrans with excellent enantioselectivity [59] (Scheme 3a). This method was successfully applied for the efficient synthesis of the commercialized fragrances Verdirosa and Pelargene. Doremox, a rose oxide replacement fragrance, can be also obtained via the diastereoselective hydrogenation of Verdirosa. More importantly, this method enables researchers to build a library of diverse and enantiomerically enriched dihydropyrans and tetrahydropyrans in a modular fashion, which may accelerate the discovery of new fragrances and promote olfactory understanding of this scaffold.

With a time-honored and worldwide utilization in a range of perfumes, including Chanel N° 5, vetiver oil represents one of the most well-known perfumery materials, but the molecular origin of its special odor remains unclear. In 2021, the List group elucidated the fragrant principle of vetiver oil in collaboration with the Kraft group at Givaudan [60]. With total synthesis and olfactory study of (+)-2-*epi*-ziza-6(13)en-3-one (Scheme 3b), they proved this is the key scent contributor to the characteristic transparent woody–ambery odor of vetiver oil. This compound has a featured and potent vetiver odor with a remarkable threshold of 29 pg/L air. Notably, its structure superimposes quite well on that of arborone in stereochemical superposition analysis, which partly revealed the molecular mechanism of their similar transparent woody–ambery notes and the “magic quasi-pheromone-like” effect.

The synthetic route was initiated by an asymmetric Mukaiyama–Michael addition [61], which is catalyzed by the IDPi on a multigram scale with an impressive 0.1 mol% of catalyst loading. After establishing the original enantioselectivity, the adduct was transformed into an enyne intermediate in several steps. The following intramolecular [2 + 2 + 1] cycloaddition is another key step, which was expected to form the desired polycyclic scaffold with sterically well-arranged substituents. However, this challenging cyclization cannot proceed under common Pauson–Khand conditions. With the compromise of atom and step economies, a stepwise protocol was finally employed. Stoichiometric Co_2_(CO)_8_ was needed to form an isolatable cobalt complex that was then treated with excess *tert*-butyl (methyl)sulfane to furnish the cyclization. Although the yield and selectivity were not high, the Pauson–Khand reaction accomplished the goal of rapid construction of the challenging scaffold; the entire synthetic route is still efficient enough when compared to the isolation of a single enantiopure ingredient from hundreds of sesquiterpenoid constituents in vetiver oil.

**Scheme 4:**
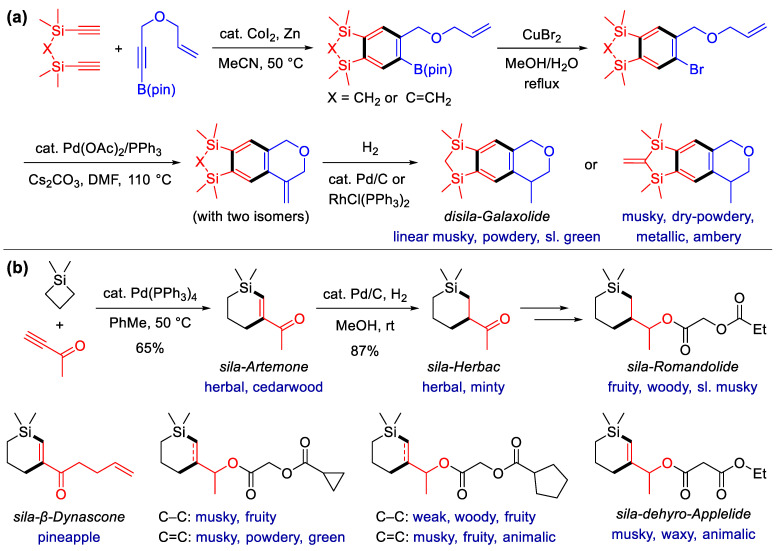
Synthesis of *Si*-heterocyclic fragrances via alkyne annulation. (**a**) Synthesis of sila-analogs of galaxolide with Co-catalyzed [2 + 2 + 2] alkyne cycloaddition as the key step; (**b**) synthesis of fragrant six-membered silacycles via the Pd-catalyzed [4 + 2] reaction of silacyclobutanes and alkynes.

Musky fragrances are omnipresent and indispensable in perfumery and exploring new types of musks is a continuous goal in fragrance chemistry. In 2013, Tacke and Kraft et al. synthesized the silicon-containing analogs of galaxolide [62] (Scheme 4a), a polycyclic musk odorant for more than half a century. A Co-catalyzed [2 + 2 + 2] cycloaddition of the disila-diynes with a multifunctional alkyne was first conducted to construct a benzene ring with well-arranged functional groups, a strategy previously used for the construction of several disila-analogs of the well-known musk, phantolide [63]. After the conversion of the borate group to bromide, an intramolecular Heck cross-coupling reaction was performed. The desired products with an exocyclic double bond were obtained, together with two separatable regioisomers; then, they underwent hydrogenation to deliver sila-analogs of galaxolide. Furthermore, the corresponding diastereomers can be obtained from the racemates by the formation–decomplexation of chromatographically separatable Cr-complexes. This work exemplified the sequential transformation of alkynes and alkenes to enable the synthesis of polycyclic fragrances, but selectivity and step economy still have plenty of room for improvement. An enantioselective reductive Heck reaction may be a possible strategy to meet this challenge [64,65].

In 2014, Liu et al. reported an efficient and regioselective Pd(0)-catalyzed [4 + 2] cyclization for the construction of a six-membered silacycle from silacyclobutanes and terminal alkynes [66] (Scheme 4b). The reaction is proposed to be initiated by the oxidative insertion of the Pd(0) species to the silacyclobutane ring, to form a five-membered palladasilacycle, which was coordinated with alkyne, with regioselectivity controlled by steric hindrance. After alkyne insertion, a seven-membered palladasilacycle is formed, which undergoes reductive elimination to yield the resulting product. This method was successfully applied for the synthesis of sila-analogs of Artemone, Herbac and *β*-Dynascone for commercial fragrances produced by Givaudan, IFF and Firmenich, respectively. Sila-Artemone was obtained first and was then converted to Sila-Herbac via hydrogenation. Sila-*β*-Dynascone can be synthesized either via the derivation of sila-Artemone, or via direct cyclization using hept-6-en-1-yn-3-one as the alkyne partner.

In a subsequent work in 2016, Liu et al. further synthesized a series of ester-containing silacycles starting from sila-Artemone [67]. Besides the sila-analogs of linear alicyclic musks such as Rosamusk, Romandolide and Applelide, analogs with cyclopropyl or cyclopentyl were also synthesized (Scheme 4b). Notably, some of the sila-analogs have a dominant fruity and woody odor instead of a musky character. After studying the odor character, thresholds and activity value, the structure–odor correlations of these *Si*-containing odorants were also analyzed. On this basis, a refined musk olfactophore model was proposed, which indicates that linear and macrocyclic musks may address the same odorant receptors.

**Scheme 5:**
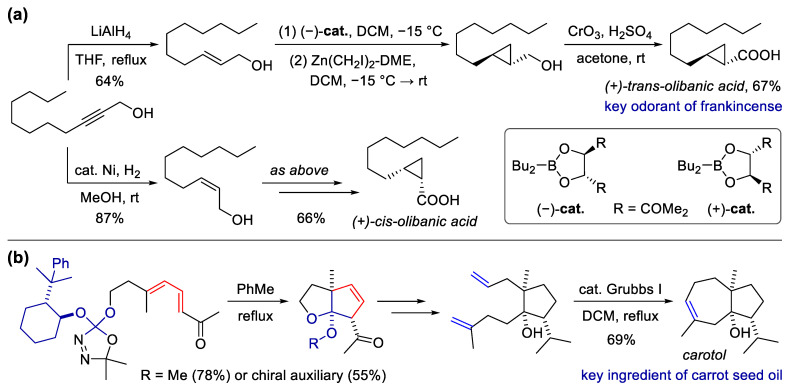
Synthesis of fragrant natural products using carbene-involved cyclization. (**a**) Total synthesis of olibanic acids via semi-hydrogenation and cyclopropanation; (**b**) total synthesis of carotol via [4 + 1] cyclization and ring-closing metathesis.

Besides π reactants and strained rings, carbenes are also a widely used component of cyclization. The cyclopropanation of alkenes using carbenes, which can be treated as a type of [2 + 1] cyclization, has been widely used for the synthesis of natural products and fragrances [31,63,68,69,70,71]. In 2016, Baldovini et al. reported an elegant total synthesis of four isomers of olibanic acids, with the key steps involved in the cis/trans-selective semi-hydrogenation of alkynes and stereodivergent asymmetric cyclopropanation [69] (Scheme 5a). Facilitated by this synthesis, they further revealed that two of them are key odorants responsible for the characteristic notes of frankincense. Carbenes can also be reacted with dienes to facilitate [4 + 1] cyclization. A representative example was demonstrated by Spino et al. for the total synthesis of carotol [72], a sesquiterpene identified as one of the major ingredients in carrot seed essential oil [73,74]. The synthetic route was initiated with an intramolecular formal [4 + 1] cycloaddition of the dialkoxycarbene and electron-deficient diene, which can also proceed smoothly using a substrate with a chiral auxiliary (Scheme 5b). After several steps involving ring-opening, functionalization and transformations, a ring-closing olefin metathesis was processed to yield carotol.

## 4. Synthesis of Fragrances via Ring-Closing Metathesis

Ring-closing metathesis (RCM) is a versatile strategy for the construction of various nonaromatic ring systems from linear substrates with two or more telechelic double or triple bonds, as has just been illustrated in the above example (Scheme 5b). While a set of classical examples of the synthesis of macrocyclic musks via olefin metathesis has been introduced elsewhere in a review [35], we would like to highlight some recent trends and unique examples on this topic and discuss other types of metathesis for the synthesis of fragrances.

Facilitated by highly active Ru catalysts and an integrated reaction–distillation strategy, Grela et al. recently developed an efficient and practical protocol to conduct RCM at high concentrations in cheap and nonvolatile paraffin oil [75]. Macrocyclic musks, including civetone and an Exaltolide analog, were successfully synthesized at a concentration of 0.2 M. Notably, the corresponding diene substrates can be easily prepared from renewable methyl oleate. In 2021, Maurya et al. reported the use of olive oil as a renewable resource for the construction of a range of 12- to 29-membered lactones and dilactones [76], further demonstrating a trend of sustainable RCM using biomass-derived building blocks [77,78,79].

Flow chemistry is another strategy to improve efficiency and productivity that has attracted increasing attention in metathesis and the fragrance industry [80,81,82,83,84]. In 2019, Collins et al. investigated the synthesis of macrocyclic musks via RCM, featuring the systematic evaluation of batch and continuous flow protocols [82]. The batch process can proceed at room temperature with 57% yield, but a rather long time period (5 days) was required. While the yield is lower (32%) and scope is limited, the continuous flow protocol, performed at 150 °C over a 5-min period, enables a higher throughput (1 g/4.8 h), which is important for large-scale synthesis. In 2021, Browne and Mauduit reported the first *Z*-selective olefin metathesis in a continuous flow, facilitated by a combination of sophisticated Ru catalysts with NHC ligands and the rational design of a continuous reactor setup [84]. By using this protocol, the highly efficient synthesis of (*Z*)-civetone and several analogs was achieved.

**Scheme 6:**
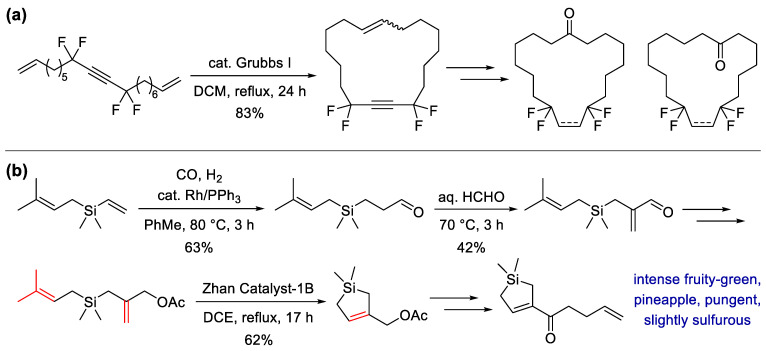
Application of olefin RCM for fragrance chemistry. (**a**) Synthesis of macrocyclic civetone analogs containing two CF_2_ groups; (**b**) synthesis of a *Si*-heterocyclic fragrance via hydroformylation and olefin metathesis.

Besides the reaction selectivity, structural diversity is also of increasing interest for the application of an RCM strategy. In 2016, O’Hagan et al. reported the synthesis of a series of fluorinated musk fragrances via olefin metathesis and post-functionalization [85,86]. From a multifunctional substrate with two terminal alkenes, an internal alkyne and two CF_2_ groups, tetrafluorinated civetone analogs were obtained (Scheme 6a). Several other civetone and muscone analogs with one or more CF_2_ groups were also synthesized. X-ray structures revealed that the CF_2_ groups had a remarkable preference for locating at the corners of these macrocycles, which greatly influence their conformations and odor, together with other factors such as their double-bond configuration and volatility. This work provides new insights on conformations and the structure–odor relationships of macrocyclic musks; the CF_2_ analog strategy may also be extended to the structure–odor studies of other types of odorants [87].

Besides carbocycles, olefin metathesis can also be used for the construction of other types of rings, using substrates with heteroatom-containing tethers. In 2014, Tacke, Kraft et al. reported a *Si*-heterocyclic fragrance with an intense fruity-green odor and an impressive odor threshold of 0.085 ng/L air, which was the lowest achieved among all the sila-odorants at that time [88]. The five-membered silacycle was constructed via RCM using the Zhan catalyst, while, formerly, Rh-catalyzed hydroformylation was another key step for simultaneously increasing the carbon chain and introducing a functional group (Scheme 6b). This route can be also used for the synthesis of its carbocyclic parent, namely, *nor*-*α*-galbanone, a fragrance with a rather low threshold of 0.0087 ng/L air. In 2017, Lovchik and Kraft further extended the RCM strategy to construct an analog of spiro [4.5]-*δ*-damascone, with silicon as the spiro atom [89].

**Scheme 7:**
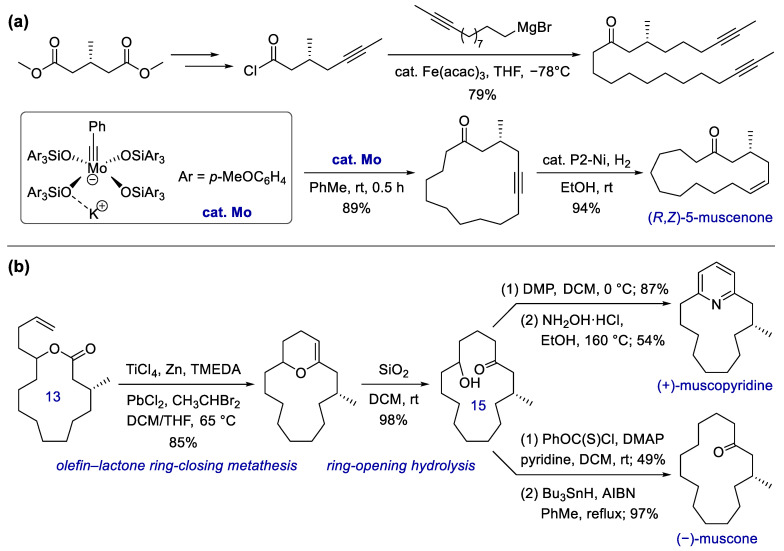
Synthesis of macrocyclic fragrances via RCM. (**a**) Total synthesis of (*R*,*Z*)-5-muscenone via coupling, alkyne RCM and semi-hydrogenation. (**b**) Collective synthesis of (+)-muscopyridine and (−)-muscone, using olefin–lactone RCM as the key step. DMP: Dess–Martin periodinane.

One bottleneck for olefin RCM is the difficulty in controlling the configuration of the formed double bond, from which a mixture of *Z*/*E* isomers is usually obtained. Alkyne metathesis is an alternative strategy, with the advantage of the synthesis of cycloalkenes with specific and predictable configurations via selective semi- hydrogenation, which has also been used for fragrance synthesis, as pioneered by the Fürstner group [90,91]. In 2012, they further developed a selective synthesis of the musk (*R*,*Z*)-5-muscenone [92] (Scheme 7a). The diyne substrate was synthesized via an Fe-catalyzed cross-coupling and subjected to the alkyne RCM procedure, catalyzed by a Mo carbyne complex. For the final semi-hydrogenation step, a P-2 Ni catalyst that was freshly prepared from Ni(OAc)_2_ and NaBH_4_ was found to be more efficient and reliable than the previously employed Lindlar catalyst. In 2014, Mathys and Kraft employed alkyne RCM and subsequent semi-hydrogenation for the selective synthesis of Aurelione [93]. Both the *Z* and *E* isomers of Aurelione have a significant musky note with different nuances. Other types of metatheses are also used for fragrance synthesis. For example, Rainier et al. developed a unique ring-closing–expansion strategy via Ti-mediated olefin–lactone RCM and subsequent ring-opening hydrolysis, applied successfully for the collective synthesis of (+)-muscopyridine and (−)-muscone from a common intermediate [94,95] (Scheme 7b). Muscopyridine was originally isolated from crude musk, which can accentuate the animalic character of musk. Muscopyridine can serve as a characteristic component for distinguishing natural and artificial musk samples by gas chromatography–mass spectrometry (GC–MS).

## 5. Synthesis of Fragrances via Rearrangement or Isomerization

The rearrangement reactions of alkenes and alkynes have been used for the synthesis of both acyclic and cyclic fragrances [96,97,98,99], and may be included as the origin of some natural fragrances [100]. As a classic example, ionones can be synthesized via acid-promoted carbocation rearrangement and cyclization from the polyene precursor pseudoionone, which can be easily obtained via the condensation of citral and acetone [52]. The main product differed in the reaction conditions. When H_3_PO_4_, H_2_SO_4_ and BF_3_·OEt_2_ were employed as the acids, *α*-ionone, *β*-ionone and *γ*-ionone were obtained as the main products, respectively. This type of annulative rearrangement was also widely used for other carbocyclic fragrances, such as Iso E Super, Iso E Super Plus and Georgywood [33], as shown in Scheme 1a.

**Scheme 8:**
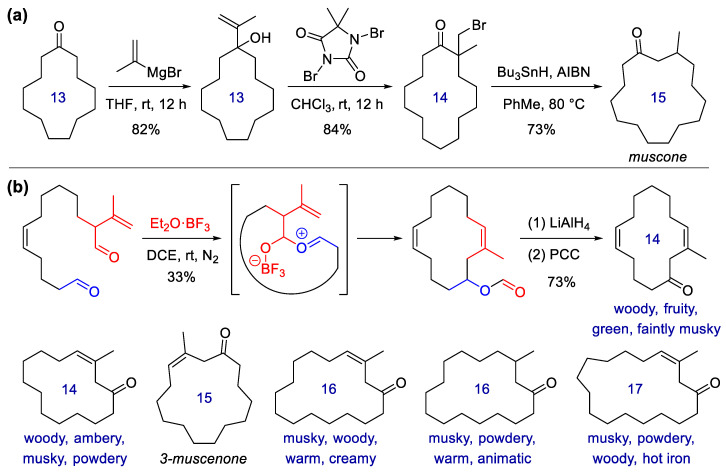
Synthesis of macrocyclic fragrances via rearrangement. (**a**) Synthesis of a muscone via a successive ring-expansion strategy; (**b**) Synthesis of diverse macrocyclic ketones via an intramolecular coupling–rearrangement cascade.

As a topologically different and complementary strategy with the ring closure of linear substrates, a ring expansion strategy is also widely used for the construction of macrocycles from more readily available substrates with smaller rings. Compared to the ring-opening hydrolysis shown in the above example (Scheme 7b), ring expansion through rearrangement is a more common procedure. In 2017, Liu and Yeung reported a successive ring expansion strategy for the construction of macrocyclic ketones [101] (Scheme 8a). First, the ketone-derived allyl alcohol substrate triggered a bromide-mediated electrophilic semipinacol rearrangement to conduct the first ring expansion. The bromide then served as a handle for subsequent radical isomerization to achieve the second ring expansion. By using this protocol, muscone and several other macrocyclic ketones were synthesized; the obtained ketones can further serve as substrates for another ring expansion sequence.

In 2011, Wang et al. developed a unique Lewis acid-promoted coupling rearrangement for the construction of a series of macrocyclic musks from multifunctional linear substrates (Scheme 8b) [102]. Two mechanisms were proposed for the rearrangement; the *oxy*-*oxonia*-Cope pathway is more favorable than the intramolecular Prins pathway, as indicated by density functional theory calculations and experimental observations. Besides 3-muscenone and muscone, several analogs were also obtained. Among them, 16- or 17-membered cyclic ketones maintain the evident musky note, but the overall characters differ in their structure. The 18-membered analog is nearly odorless, indicating that the ring size is crucial to achieving the musky odor. Notably, a 14-membered analog with two double bonds has a woody, fruity and green odor but very faint musk facets, indicating the (10*Z*)-double bond in this compound has a negative influence on the musky odor. By the comparison of these compounds with the typical macrocyclic and linear alicyclic musks, a musk olfactophore model was generated, providing valuable for better understanding the musk olfactory receptors.

**Scheme 9:**
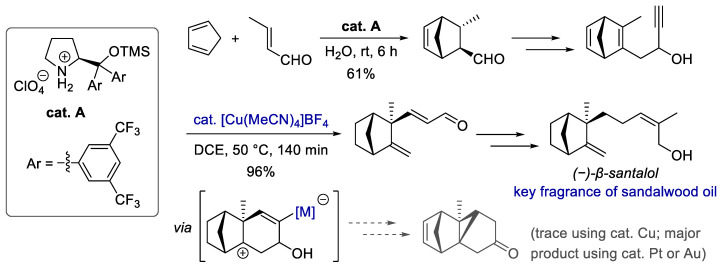
Total synthesis of (−)-*β*-santalol via asymmetric Diels–Alder reaction and Cu-catalyzed rearrangement of enynol.

As the principal fragrance of the precious sandalwood oil, (–)-*β*-santalol has represented an attractive target for chemists for many years [103]. In the first enantioselective total synthesis of (−)-*β*-santalol, accomplished by Fehr et al. from Firmenich, a unique Cu-catalyzed rearrangement was incorporated [104] (Scheme 9). The synthetic route began with an asymmetric and *exo*-selective Diels–Alder reaction via an iminium ion activation strategy developed by MacMillan et al. [105] and a chiral amine catalytic system developed by Hayashi et al. [106]. The adduct was then converted to an enynol intermediate, which underwent a Cu-catalyzed cyclization–fragmentation sequence to yield the desired aldehyde product. Notably, the rearrangement pathway can be controlled by the catalytic system. When PtCl_2_ or [PPh_3_AuCl]/AgBF_4_ was employed, a polycyclic ketone was obtained as the major product, via a more complex pathway involving cyclopropanation. Based on this work, Fehr et al. further improved the synthesis of (−)-*β*-santalol [107], while their colleagues, Chapuis et al., performed a systematic study of stereoselectivity and the mechanism of related Diels–Alder reactions [108]. Fehr et al. also investigated the synthesis of thujopsanone-related molecules via Au- or Cu-catalyzed cycloisomerization [109].

**Scheme 10:**
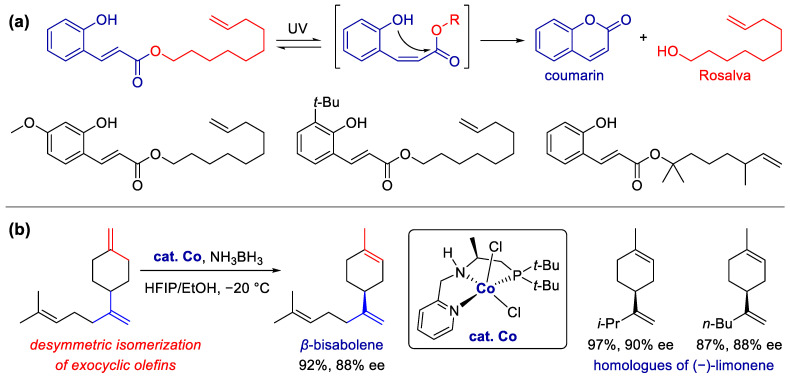
Double bond isomerization for the generation of fragrances. (**a**) UV-induced alkene isomerization of *o*-hydroxy cinnamates to release coumarin and fragrant alcohols; (**b**) asymmetric synthesis of *β*-bisabolene via Co-catalyzed desymmetric isomerization.

The isomerization of carbon-carbon double bonds has been used in the synthesis of fragrances with either linear or cyclic scaffolds [79,110,111,112]. Flachsmann et al., from Givaudan, have devised an interesting strategy for the controlled release of perfume under ambient light [113]. They synthesized several *o*-hydroxy cinnamates and found that they undergo a UV-induced alkene isomerization to trigger intramolecular transesterification and form the coumarin ring (Scheme 10a). An alcohol was also released simultaneously; this can be chosen from a library of fragrant alcohols, such as Rosalva, a widely used fragrance with fresh and floral rose notes. They further combined this strategy with the lipase-catalyzed cleavage of carbonates to achieve the controlled release of ternary fragrances [114]. In these cases, *o*-hydroxy cinnamates can be treated as profragrances (similar to prodrugs) to achieve a long-lasting perfume practically [115,116].

In 2021, Liu developed a desymmetric isomerization strategy to synthesize chiral cyclohexene derivatives from exocyclic olefins [117] (Scheme 10b). They designed and prepared a set of chiral NNP-pincer cobalt catalysts for this transformation and employed it for the asymmetric synthesis of *β*-bisabolene, a fragrant sesquiterpene found in many essential oils that has antitumor activity. The scope of this reaction is rather broad, encompassing chiral cyclohexenes with various substituents, including those treated as homologs of (−)-limonene. Although odor studies were not performed for these products, they may be useful to solve the fragrance mystery of limonene and the related terpenoids [118].

## 6. Synthesis of Heterocyclic Fragrances via Addition-Type Reactions

The addition of unsaturated hydrocarbons is part of a large group of reactions with broad application in the fragrance industry. Many achievements have been made in the synthesis of acyclic and carbocyclic fragrances via addition-type reactions, including recent progress using asymmetric addition and hydrogenation for fragrances [119,120,121,122,123,124,125]. In this section, we would like to highlight one undervalued direction, using addition-type reactions for heterocyclic fragrances. Synthesis involving addition or formal addition as a key step will be discussed, including hydroalkoxylation, the Prins reaction, hydroformylation and hydroarylation, as well as the related multi-step transformations and cascade reactions.

**Scheme 11:**
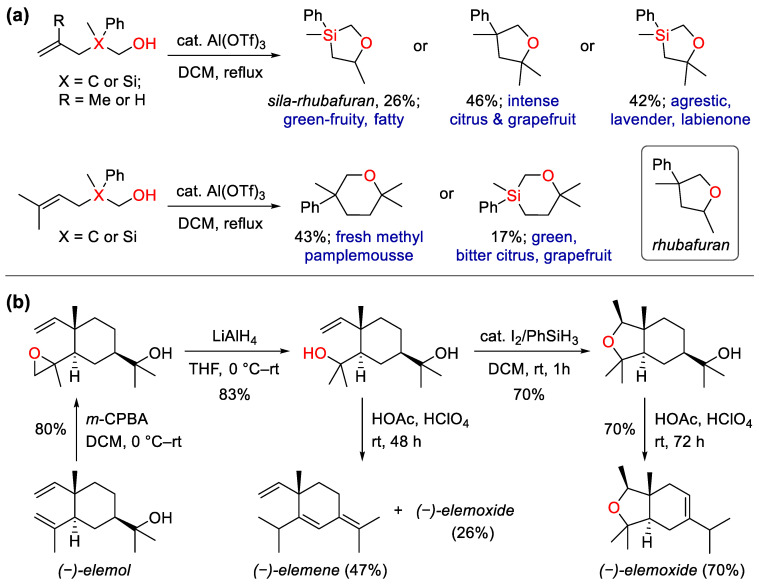
Synthesis of heterocyclic fragrances via intramolecular hydroalkoxylation. (**a**) Synthesis of the silicon-containing and ring-expanded analogs of rhubafuran. (**b**) Synthesis of (−)-elemoxide from (−)-elemol. *m*-CPBA: *m*-chloroperbenzoic acid.

The intramolecular hydroalkoxylation of linear alkene–alcohol substrates represents a general strategy for the construction of *O*-heterocycles, in which additional heteroatoms can be introduced to the tether between the two functional groups. With their continued interest in *Si*-containing fragrances, the Tacke group employed Lewis acid-catalyzed hydroalkoxylation for the synthesis of several analogs of rhubafuran with green and fruity notes [126] (Scheme 11a). A five-membered ring was formed when terminal alkenes were used, while substrates with internal double bonds led to a six-membered ring. By introducing a silicon atom to the tether, sila-rhubafuran and other *Si*-heterocyclic fragrances can be efficiently constructed. 

In 2018, Majik et al. reported a semi-synthesis of (−)-elemoxide [127] (Scheme 11b), a sesquiterpene oxide with scents of rhubarb, laurel and thyme. The route of synthesis started with the regioselective epoxidation of natural extracted (−)-elemol, with subsequent reductive ring-opening. An intramolecular hydroalkoxylation then followed, under the I_2_/silane catalytic system, to construct the tetrahydrofuran ring. Finally, elemoxide was obtained via dehydration, with a 32% overall yield in four steps. Direct treatment of the diol intermediate under acid conditions produced (−)elemene as the major product, together with elemoxide. Considering that elemoxide is actually an isomer of elemol, a direct rearrangement with ideal atom, step and redox economies may be possible. Thus, this transformation could serve as an interesting test ground for the development of new catalytic systems for a selective dehydration–hydrolysis cascade.

**Scheme 12:**
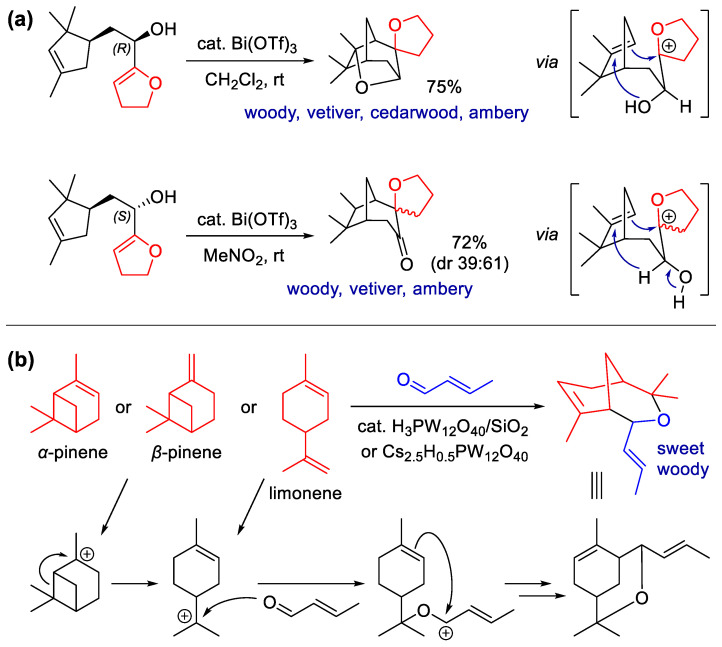
Synthesis of bridged *O*-heterocyclic fragrances involving carbocation intermediates. (**a**) Bi-catalyzed cyclization of campholenal-derived enol ethers. (**b**) Heteropoly acid-catalyzed cyclization of crotonaldehyde with limonene or pinene.

The synthesis of fragrances using the renewable building blocks in essential oils represents a time-honored and still thriving strategy for biomass upgrading. Campholenal, a widely occurring, natural monoterpenic aldehyde has been employed for the synthesis of various commercialized sandalwood-scented fragrances such as Polysantol, Javanol and Dartanol (Bacdanol). In 2018, Filippi and Lemière et al. reported the Bi-catalyzed stereocontrolled reactions of campholenal-derived enol ethers [128] (Scheme 12a). By using the (*R*,*R*)-isomer, a tetracyclic spirodiether was constructed via a cascade cyclization mode that they had reported previously [129,130]. When the (*R*,*S*)-isomer was reacted in nitromethane, a tricyclic spiroketone was formed. This method was then extended to construct a library of complex spiro polyheterocycles, some of which have woody and vetiver-like scents. After investigation of their relative odor strengths with an enantioselective GC–MS–olfactometer equipped with a *γ*-cyclodextrin-derived selector as the stationary phase, the analysis revealed that only spiroxides derived from (*R*)-campholenal were vetiver-scented, while those from (*S*)-campholenal were odorless.

In the past decade, Gusevskaya and coworkers developed a series of catalytic transformations of renewable alkenes to gain access to new fragrant molecules [30,131,132,133,134,135,136,137,138]. For example, they subjected myrtenol and nopol to the synthesis of polycyclic fragrances via hydroformylation and the subsequent intramolecular acetalization [131]. In 2020, they reported a hydroformylation protocol in eco-benign solvents to produce new woody fragrances from natural caryophyllane sesquiterpenes [132]. Previously, they discovered a unique bridged *O*-heterocycle with a sweet, woody odor by the heteropoly acid-catalyzed cascade coupling/cyclization of limonene and crotonaldehyde [133] (Scheme 12b). *α*-Pinene and *β*-pinene are also reactive with an additional carbocation rearrangement, indicating that the nucleophilic attack of aldehyde on the protonated alkenes may be more favorable than the nucleophilic attack of alkene on the protonated aldehyde. They also developed a cascade hydroformylation/cyclization protocol for the direct conversion of limonene to a polycyclic alcohol used for perfumes [134]. By using a combined catalytic system, the reaction proceeds smoothly via Rh-catalyzed hydroformylation and the cascade intramolecular Prins reaction catalyzed by pyridinium *p*-toluenesulfonate.

The Prins reaction, also known as the carbonyl–ene reaction, is an acid-catalyzed addition of alkenes with aldehydes that is widely used for the synthesis of fragrant alcohols and esters [32]. Interestingly, the discovery of this type of reaction can be traced back before Prins’s systematic study. In 1899, O. Kriewitz reported the thermal addition of *β*-pinene with formaldehyde to yield nopol, a fragrance found in some essential oils that is used to add a woody scent to soaps and detergents. Nopol can be also used as a pesticide and it is a building block for drugs such as pinaverium.

**Scheme 13:**
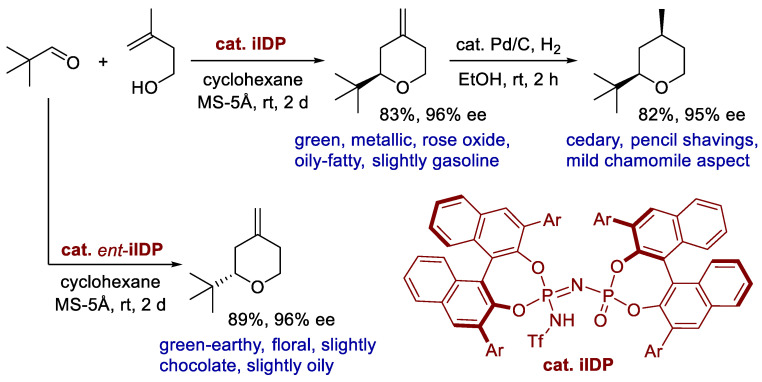
Synthesis of *O*-heterocyclic fragrances via an enantioselective Prins reaction catalyzed by imino-imidodiphosphate (iIDP).

In 2016, the List group reported a general synthesis of chiral tetrahydropyrans, catalyzed by confined imino-imidodiphosphate (iIDP) Brønsted acid [139] (Scheme 13). The reaction begined with an enantioselective intermolecular Prins reaction of homoallylic alcohols with aldehydes, followed by intramolecular ester formation. Notably, a stereodivergent synthesis was achieved by using iIDP and *ent*-iDP catalysts, to yield both enantiomers up to gram scale and 96% ee. By using this cyclization and the subsequent hydrogenation of the exocyclic double bonds, fragrances including rose oxide, Doremox and a series of analogs can be synthesized. As illustrated by the example shown in Scheme 13, both the chiral center and the double bond have a crucial impact on the odor character and threshold. This efficient and enantioselective method enables modular synthesis for hydropyrans. By combining this with the work shown in Scheme 3a, a large-membered library can be built, founding a molecular basis to further explore the structure-odor relationship of *O*-heterocyclic fragrances.

**Scheme 14:**
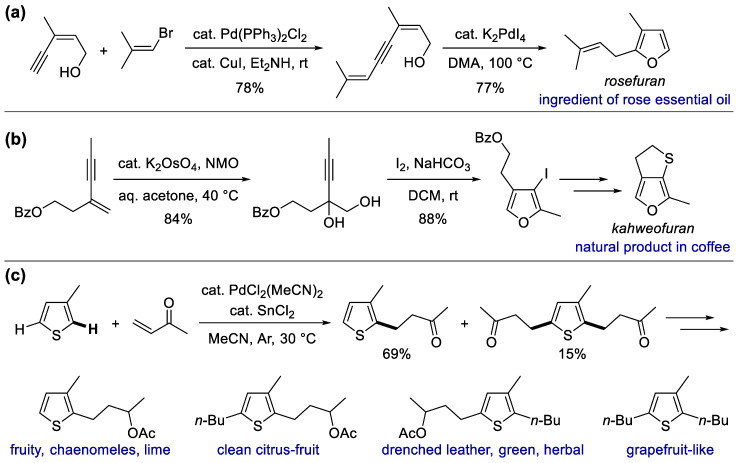
Synthesis of multisubstituted furans or thiophenes via the addition of unsaturated bonds. (**a**) Synthesis of rosefuran via Pd-catalyzed reaction. (**b**) The key cyclization step for synthesis of kahweofuran; NMO: *N*-methylmorpholine oxide. (**c**) Synthesis of fragrant thiophene derivatives, using C–H functionalization as a key step.

While the intramolecular hydroalkoxylation of alkenes is widely used for the construction of non-aromatic *O*-heterocycles, the hydroalkoxylation of alkynes and enynes is widely used for aromatic *O*-heterocycles such as furans. A classical example was reported by Gabriele and Salerno, in which the rapid assembly of rosefuran was achieved by Pd-catalyzed cycloisomerization, initiated by the intramolecular nucleophilic attack of the hydroxy group on the alkyne [140] (Scheme 14a). This protocol can also be extended for a range of multi-substituted furans [141].

In 2021, King and Knight reported the total synthesis of kahweofuran, a small but unique fused heterocycle isolated from coffee extract. The key furan formation step was an iodine-mediated cyclization of the alkyne–diol substrate, which was prepared via successive transformations of carbon-carbon multiple bonds [142] (Scheme 14b). When kahweofuran was studied previously, it was found to have a “violent, sulfury” odor but “a pleasant roasted and smoky note” at highly diluted concentrations [143]. Thus, kahweofuran was considered to be one of the key ingredients for coffee aroma and attracted several groups interested in developing its synthetic procedures. Unexpectedly, the synthetic kahweofuran produced by this route was essentially odorless; the authors suggested that the odor of the previously isolated kahweofuran may have originated from some thiol-containing impurities.

In the past two decades, the direct C–H functionalization of aromatic heterocycles has been widely explored for rapid access to functionalized rings from simple substrates, which process is strategically different from cyclization. With their continuing interest in thiophene fragrances [144], Kula et al. recently synthesized several multi-substituted thiophene fragrances with long-lasting citrusy odor via the C–H functionalization of 3-methylthiophene with methylvinylketone and several subsequent steps of derivation [145] (Scheme 13c). The C–H addition to alkenes can either be promoted by BF_3_ or catalyzed by a Pd/Sn co-catalytic system [146].

## 7. Synthesis of Fragrances via the Cascade Cyclization of Enynes

The cascade cyclization of multifunctional linear substrates is a step-economic way to gain access to chemical complexity from simplicity, which is a maneuverable strategy as part of Nature’s synthetic power for synthesis of fragrances. For example, the biosynthesis of terpenoids and their derivatives, such as santalol and sclareol, was deemed to include a cationic cascade cyclization and the rearrangement of a linear polyene precursor, which can also be achieved in microbial cell factories [103,147,148,149,150,151]. Since Johnson’s landmark synthesis of progesterone [152], the biomimetic domino cyclization of polyenes and enynes has emerged as one of the most powerful strategies for the construction of polycyclic scaffolds [153].

**Scheme 15:**
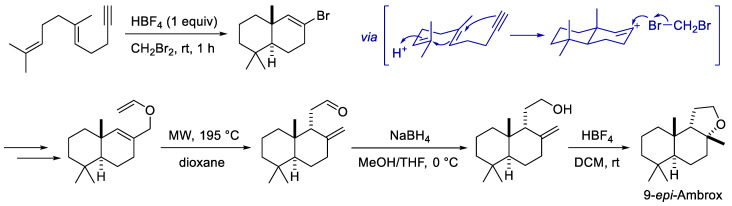
Total synthesis of 9-*epi*-Ambrox initiated with a cascade cyclization of dienyne.

Ambergris is a kind of excretion from sperm whales and has been considered one of the most precious perfume materials for centuries. As the odor principle of ambergris, Ambrox is used to endow a delicate base note on a range of perfumes and serves as a prototype to develop other ambergris-type fragrances [29]. In 2018, Eichhorn et al. developed an industrial-scale biotransformation process for producing (−)-Ambrox from (*E*,*E*)-homofarnesol via cascade cyclization, catalyzed by an improved squalene hopene cyclase enzyme in *Escherichia coli* cells [154]. In 2016, Fañanás and Rodríguez developed an efficient and practical total synthesis of 9-*epi*-Ambrox from a dienyne substrate that is readily available from cheap and renewable geraniol [155] (Scheme 15). The key step was the acid-promoted cationic cyclization of the dienyne with tandem nucleophilic bromination of the formed carbocationic intermediate, assembling the carbocyclic skeleton with a bromide handle for subsequent transformations. After the installation of an enol ether group, a microwave-accelerated Claisen rearrangement was conducted, followed by reduction and intramolecular addition to construct the fused tetrahydrofuran ring. Notably, only one purification process was needed for the seven steps, and the final product can be obtained at gram scale with a 35% overall yield.

**Scheme 16:**
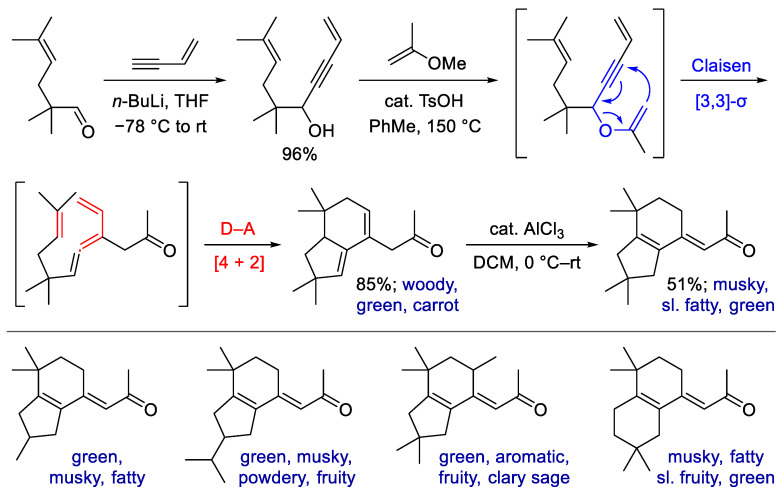
Synthesis of bicyclic musks via the cascade pericyclic reaction of the multifunctional enyne substrates.

In 2020, Wang and coworkers developed a unique synthesis of a set of bicyclic dienone fragrances with musky and green notes [156]. First, the enyneol substrate was prepared via the alkylation of aldehydes, and then underwent a Saucy–Marbet rearrangement with isopropenyl methyl ether (Scheme 16). A cascade pericyclic reaction consisting of a [3,3]-sigmatropic rearrangement and an intramolecular Diels–Alder reaction was conducted. The obtained ketone has a woody, green and carroty scent, and it can be isomerized under Lewis acid conditions to form a conjugated dienone product with a significantly musky character. By tuning the substituents, a series of analogs were obtained. By using quantum mechanics/molecular mechanics calculations, these dienone musks were predicted in silico to bind to the olfactory receptor OR5AN1; however, they were inactive in the in vitro assay. Musks with animalic and powdery notes, such as muscone and musk ketone, can strongly activate the receptor OR5AN1, while other types of musks do not show such significant activity. On this basis, the authors proposed that OR5AN1 may be responsible for the animalic character rather than the musk character, and the prime musk receptor still needs to be discovered. Due to the combinatorial strategy found in the olfactory system, this musky odor may be related to several receptors. To further illuminate this issue, the discovery, synthesis and biological studies of new musks, especially those with new scaffolds, is a lasting goal in fragrance chemistry.

**Scheme 17:**
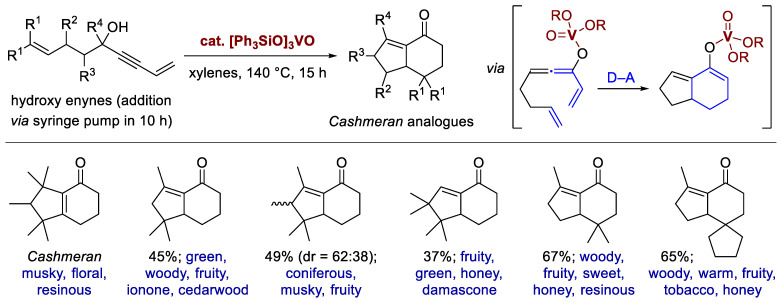
Synthesis of Cashmeran analogs via the oxovanadium-catalyzed cascade cyclization of hydroxy enynes.

Using a similar cascade cyclization strategy, Wang et al. also achieved the efficient construction of bicyclic fragrances with a Cashmeran-like scaffold via the oxovanadium- catalyzed cycloisomerization of enyneols, with 100% atom economy [157] (Scheme 17). The cyclization is surmised to occur via a tandem catalytic Saucy–Marbet-type rearrangement and thermal intramolecular Diels–Alder reaction. Although showing a similar bicyclic scaffold, only one product has a musky note, while the others usually have fruity and woody odors, indicating that their 3D structures may have changed with the different positions of double bonds and the variations in substituents. The use of these compounds in perfumery is limited due to their poor biodegradability, but they may be used for further structure–odor relationship study of Cashmeran-like scaffolds and serve as molecular probes for olfactory receptors.

**Scheme 18:**
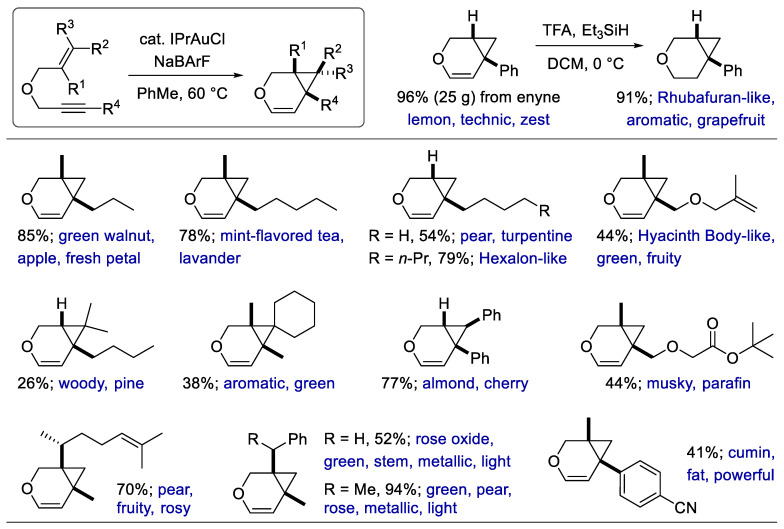
Synthesis of bicyclic fragrances from oxygen-tethered enynes via gold-catalyzed cycloisomerization.

In 2020, Michelet et al. reported a robust protocol for rapid assembly of 3/6-membered fused rings via gold-catalyzed cycloisomerization of oxygen-tethered enynes [158] (Scheme 18). Remarkably, the reaction can be performed on a 25 g scale with 0.04 mol% catalyst loading in mild conditions, which highlights the practicality of the reaction. A library of bicyclic molecules was synthesized and their olfactory properties were examined. The odor characteristics of these compounds are highly diversified, depending on the substituents, and several of them have scents related to several well-known synthetic perfumes, including Rhubafuran, Hexalon and Hyacinth Body. Very recently, they further expanded the scope of the reaction to enrich this new class of fragrances [159]. Several sulfur-tethered enynes were also tested for this cycloisomerization process but they were totally inactive. Interestingly, the group found that most of the enyne substrates also had significant odors, among them the enynes and their annulative products, which usually have fruity, green and floral notes with a pear-like scent.

## 8. Conclusions

In recent years, the development of synthetic methodologies has provided more efficient, practical and sustainable avenues for the production of fragrances. With readily available alkene and alkyne building blocks, a large number of classic fragrances and new odorants have been synthesized, enriching the molecular library and odor palette for perfumers and olfactory researchers. A range of synthetic strategies and methods have been exploited, aiming for cyclic fragrances, such as cycloaddition, metal- or organo- catalyzed intermolecular cyclization, ring-closing metathesis, intramolecular addition and rearrangement reactions. The combination of several alkene/alkyne transformations in a cascade or stepwise fashion has enabled rapid access to more complex multifunctional or polycyclic fragrances. Apart from fragrant carbocycles, oxygen- and silicon-containing heterocycles have also been widely investigated, but the application of these reactions to sulfur- and nitrogen-containing fragrances still needs more exploration.

Fragrance molecules often serve as targets during the thriving of asymmetric organocatalysis and metathesis reactions; this represents a convincing and inspiring example of the collaborative development of organic chemistry and the fragrance industry. Some frontier areas, such as C–H functionalization, photocatalysis and electrosynthesis, have not paid enough attention to the field of fragrances, compared to the progress of methodologies and applications for natural products, drugs and functional molecules. With the ability of efficient coupling and cyclization, these methods are expected to have broad and fruitful applications for fragrance manufacture in the next decade [160,161,162,163,164]. Advanced technologies, such as continuous flow and automated synthetic platforms, have emerged that greatly improve efficiency and reduce carbon release during fragrance production, but the field still has much room for development. The utilization of machine learning, artificial intelligence and brain–computer interfaces for fragrance design, synthesis and quantitative olfactory studies is expected to show rapid growth [165,166,167,168,169,170,171,172,173]. The safety, therapeutic efficacy, allergic effect and biodegradability of these new fragrances should also be investigated further, especially in the case of fragrances containing new scaffolds [174,175,176,177,178,179,180].

Olfaction represents one of the most wonderful connections between animals, including humans, and Mother Nature, while these tiny and subtle fragrance molecules with structural and sensory beauty serve as linkages for chemical innovation and olfactory art. We expect that more treasures in the field of fragrance chemistry could be mined with advanced synthetic toolkits, while the pursuit of new fragrances would in turn stimulate the next blossoming of organic synthesis, olfactory chemistry and the fragrance industry. Within this broad scope, the time-honored chemistry of alkenes and alkynes will continue to play an important role in this exciting future.

## Data Availability

Not applicable.
